# *Galleria mellonella* as a Model for the Study of Fungal Pathogens: Advantages and Disadvantages

**DOI:** 10.3390/pathogens13030233

**Published:** 2024-03-07

**Authors:** Andrea Giammarino, Nicolò Bellucci, Letizia Angiolella

**Affiliations:** Department of Public Health and Infectious Diseases, Sapienza University of Rome, Piazzale Aldo Moro 5, 00100 Rome, Italy; andrea.giammarino98@gmail.com (A.G.); bellucci.nico99@gmail.com (N.B.)

**Keywords:** *Galleria mellonella*, fungi, *Aspergillus* spp., *Candida* spp., virulence factors

## Abstract

The study of pathogenicity and virulence of fungal strains, in vivo in the preclinical phase, is carried out through the use of animal models belonging to various classes of mammals (rodents, leproids, etc.). Although animals are functionally more similar to humans, these studies have some limitations in terms of ethics (animal suffering), user-friendliness, cost-effectiveness, timing (physiological response time) and logistics (need for adequately equipped laboratories). A good in vivo model must possess some optimal characteristics to be used, such as rapid growth, small size and short life cycle. For this reason, insects, such as *Galleria mellonella* (Lepidoptera), *Drosophila melanogaster* (Diptera) and *Bombyx mori* (Lepidoptera), have been widely used as alternative non-mammalian models. Due to their simplicity of use and low cost, the larvae of *G. mellonella* represent an optimal model above all to evaluate the virulence of fungal pathogens and the use of antifungal treatments (either single or in combination with biologically active compounds). A further advantage is also represented by their simple neuronal system limiting the suffering of the animal itself, their ability to survive at near-body ambient temperatures as well as the expression of proteins able to recognise combined pathogens following the three R principles (replacement, refinement and reduction). This review aims to assess the validity as well as the advantages and disadvantages of replacing mammalian classes with *G. mellonella* as an in vivo study model for preclinical experimentation.

## 1. Introduction

The experimental use of animals is extremely important in science, especially for the development of new antimicrobial drugs with enhanced safety and efficacy [1]. However, at such a preclinical phase, in vivo mammalian models, primarily mice and rats, have some disadvantages, such as the need for adequate infrastructure and lengthy experiments. In addition, they always pose a number of critical issues due to the identification and use of animal models complying with the ethical, experimental and legislative principles recommended by the European directive on animal protection guided by the three R rules (i.e., replacement, reduction and refinement) [2,3]. In recent years, however, many in vivo studies have used insects, which approximately account for 90% of all animal species. The insect immune system shares many features with human innate defence; therefore, it can be called “evolutionary roots of human innate immunity” [4]. For this reason, insects are used not only in studies of the interactions with their natural pathogens but also in studies of the virulence factors of human pathogens as well as in tests of antimicrobial drugs in vivo [5,6,7]. Therefore, insects, such as *Galleria mellonella* (Lepidoptera), *Drosophila melanogaster* (Diptera) and *Bombyx mori* (Lepidoptera), have been widely used as alternative non-mammalian models. Table 1 shows the differences among the invertebrate models utilised in fungal infections.

Thanks to its rapid life cycle, cost-effectiveness and advanced technology availability, *Drosophila melanogaster*, commonly known as the fruit fly, is a model organism used to study a wide range of disciplines, from fundamental genetics to tissue and organ development [8]. The *D. melanogaster* genome is 60% homologous to that of humans and since about 75% of the genes responsible for human diseases have homologues in flies [9], these insects have recently become useful tools for studying human diseases, including rare Mendelian diseases [10], neurodegenerative diseases [11] and cancer [12]. The molecular mechanisms of pathogenic proteins encoded by viral and bacterial genomes have also been studied in *Drosophila* [13].

*B. mori*, also known as the silkworm, is often used as an infection model. This is due to the availability of germ plasm banks, which maintain genetic stock collections; these centres adopt an artificial diet, thus contributing to standardising the quality of the supply of this insect [14]. The organs and systems of *B. mori* and mammals are anatomically similar, thus making this insect a valuable model organism for studying various life science processes. This has been made possible by the availability of the complete sequence of its genome and the development of technologies for genetic manipulation. Finally, *B. mori* is still widely used in sericulture and biotechnology as a bioreactor for producing recombinant proteins and silk-based biomaterials [15].

Alternative in vivo models, such as *Galleria mellonella*, have been studied [16,17,18,19]; moreover, this insect has been widely used in experiments to evaluate the toxic potential and antimicrobial activity of drugs, including antifungal agents [20].

*G. mellonella* is also a suitable model for studying the expression of virulence factors and host–pathogen interactions, such as the innate immune response to microorganisms, thus representing the first step in human health studies [21]. One of its most important characteristics is its innate immune system, whose functional structures are similar to those of mammals [22].

The treatment of infections caused by fungal pathogens is extremely challenging both due to the presence of MDR (multidrug-resistant) strains mainly infecting immunocompromised patients and the limited availability of antifungal drugs, which are highly toxic [23]. The most studied fungi are *Aspergillus fumigatus*, *Candida albicans* and *Cryptococcus neoformans*, which cause a high mortality rate [24,25,26,27]. Therefore, there is a need for an in vivo phase of experimentation involving the study of fungal pathogens and their pathogenic mechanisms in order to assess the host–pathogen interaction and evaluate the efficacy of treatments. This review aims to evaluate the validity, advantages and disadvantages related to substituting mammalian classes for *G. mellonella* as an in vivo study model for preclinical experimentation.

## 2. *Galleria mellonella* Model

**Figure 1:**
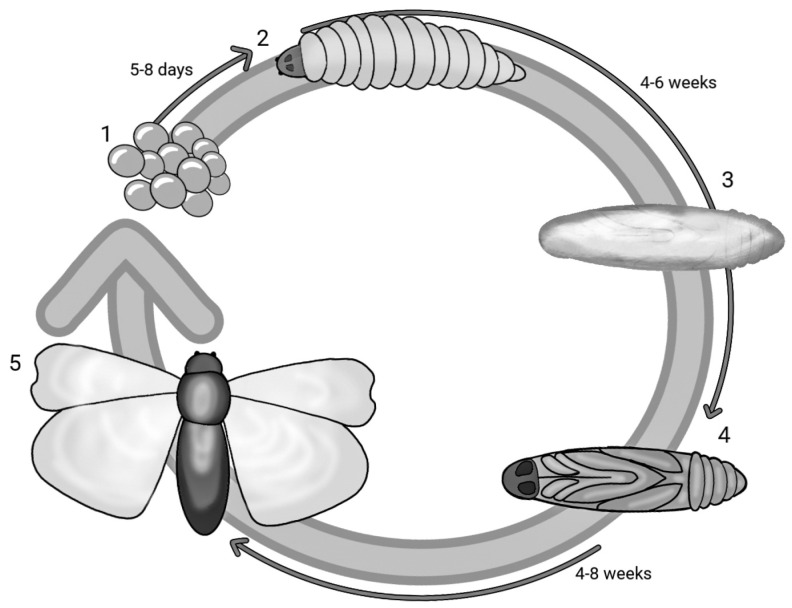
Life cycle of *Galleria mellonella*: (1) eggs; (2) caterpillar; (3) pre−pupa; (4) pupa; (5) adult form.

*G. mellonella*, a species belonging to the order Lepidoptera and part of the Pyralidae family, is a ubiquitous parasite in the hives of bees, wasps and bumblebees that feeds on honey, beeswax, bee faeces, pollen and cocoons. With a holometabolous life cycle, this insect has five stages of development and therefore a very short life cycle: egg, caterpillar, pre-pupa, pupa and adult. Figure 1 reports the life cycle of *G. mellonella* in different stages and the overall timeframe. The eggs develop into caterpillars after 5–8 days, and in about 6 weeks, the caterpillar matures and grows. The larval stage has a cylindrical elongated form measuring 16–20 mm. After 8 to 10 moults taking place from day 28 to 6 weeks, the caterpillar stops feeding and maintains slight motility, but in the meantime, the development of a silk cocoon begins—this is the pre-pupa stage [28,29]. The pre-pupa subsequently matures into a pupa, immobilised in the cocoon [30]. The adult form appears after a period ranging from 4 to 8 weeks; the adult moth has a reddish-brown colour and is active in the nocturnal phase. In its adult form, *G. mellonella* can lay up to 300 eggs, although some studies report a higher number of up to 600 [31,32,33,34,35,36]. The life cycle from the hatching of the eggs to the maturation of the larvae may be affected by nourishment and temperature (the optimal one being between 28 and 30 °C) [37]. Its quick and easy life cycle requires no special attention. Unlike other insects, the larvae of *G. mellonella* survive at temperature ranges that vary from 15 °C to 37 °C. At 37 °C, human physiological conditions can be mimicked, and this is necessary to reach a temperature at which the virulence factors of pathogens can be expressed [5,38].

The nervous system of *G. mellonella*, like all Lepidoptera, is very simple from a functional and anatomical point of view, thus enabling our group of scientists to reconstruct it using computer software (Amira 5.3.3) [39].

In the larval form, we have the following subdivisions of the body: head, three thoracic segments with two legs and an abdomen consisting of ten segments. The abdomen has eight prolegs and two anal prolegs [40].

The larval body has an internal cavity with an open circulatory system with a digestive system, which originates with a simple masticatory mouthpart (with attached salivary glands) and ends with an anal opening. Furthermore, ventrally, we find a nervous system characterised by ganglia and various neuronal connections [41]. This system makes the organism sensitive to abiotic factors such as light, temperature and humidity [39].

Sexual polymorphisms can be identified in the adult form. The male emits ultrasound as a form of mating call and is smaller and beige in colour; meanwhile, the female is larger, with a wider wingspan and releases pheromones (non-anal), useful for attracting the male [38].

Experimental research aimed at sequencing the genome of *G. mellonella* has been recently conducted, and from the analyses carried out using PacBio technologies and genomic libraries, it was realised that the DNA of larvae has a modest amount of genetic homologies with the genomes of humans, rats and other organisms. These features promote and encourage the use of Lepidoptera as a model in biomedical research [22].

## 3. *G. mellonella* Immune System

The immune system of *G. mellonella* is organised in an innate form that can be divided into humoral and cellular responses. The latter, unlike the former, presents a series of homologies with the immune systems of mammals [22], a characteristic confirmed by a series of experimental tests [42,43,44,45]. This innate immune system is the first line of defence used by the insect against various pathogens, as also seen in vertebrates [42]. The ability of the insect to exploit the cuticle is mainly due to its structure. The endocuticle, the innermost layer, contains chitin fibrils, while the outermost layer, the epicuticle, contains fatty acids, lipids and sterols [43]. This creates a dense and resistant barrier against pathogens and harmful mechanical forces, but it can be damaged or degraded [44].

Another system that participates in nonspecific immune action is the haemolymph, equivalent to mammalian blood; therefore, it is responsible for the transport of various substances (e.g., nutrients, signal molecules and waste) [45].

### 3.1. Cellular Immune System

*G. mellonella* haemocytes, such as plasmatocytes and granulocytes, due to their adherent properties, are phagocytic cells. Haemocytes are responsible for the phagocytosis, encapsulation and nodulation of the invading pathogen. Haemocytes may either be free in the haemolymph or associated with specific internal organs (especially the digestive, reproductive and cardiac ones) [46,47].

The haemocytes are able to recognise pathogens through specific receptors present on their surface: the PRRs (pathogen recognition receptors). The pathogenic antigens that are recognised by these receptor structures are PAMPs, which include lipopolysaccharides (LPS), peptidoglycans, lipoteichoic acids (LTA) and β-1,3 glucan [48]. The recognition molecules in question include apolipophorin–III (ApoLp III), which is capable of identifying fungal β-1,3 glucan as well as bacterial LPS and LPA [48]. Haemolin does not have direct antibacterial properties, although it binds to the lipoteichoic acid of Gram-positive bacteria and to the lipopolysaccharides (LPS) of Gram-negative bacteria [49].

Table 2 shows the different haemocytes and their functions [46,50].

Encapsulation comprises the development of capsules enveloping and blocking a possible no-self agent, which is internalised in the insect. Encapsulation occurs when pathogens are too large for phagocytosis. The response of the host to microbial invasion is characterised by the development of nodules, called nodulation [38]. The process starts with granular cells attacking the surface of the microbes. This triggers the release of multiple plasmatocyte-spreading peptides, attacking the surface of bacteria, fungal spores or foreign targets, resulting in the formation of a smooth capsule [47]. Melanisation does not occur in this process.

Melanisation, as the main defence mechanism against a high range of microorganisms and comprising the deposition of melanin in the haemolymph of the pathogen, will be followed by the coagulation of the haemolymph and opsonisation in order to kill the pathogen [50]. This process begins on the cuticle surface of the larvae with simple black spots, gradually spreading to the entire cuticular surface if the infection becomes more severe. This melanisation may involve the entire larva, to the point of making it completely black; and the latter condition is synonymous with a serious infection, causing the death of the larvae [39]. The melanisation process is activated by surface receptors, which recognise specific molecular patterns. Among the receptors able to recognise β-1,3-glucan, LPS and peptidoglycans, there is also C-reactive protein, a homologue of TLRs in mammals [51]. This is released and carried either to the cuticle, the damaged site or the encapsulated pathogen until the polymerisation of melanin is generated [51].

### 3.2. Humoral Immune System

The humoral immune response of *G. mellonella* involves various processes and molecular responses, which do not include antibodies (as in mammals) but rather simple antimicrobial peptides (AMPs). The immediate contact with microorganisms (bacteria, fungi, viruses, protozoa) determines the transcription of genes for the synthesis of AMPs [52].

AMPs are polypeptide chains of 10–40 residues playing a fundamental role in host defence and which are produced mainly in body fat in both the digestive and reproductive tract to be subsequently released into the haemolymph. They are produced in high concentrations in the first six hours of the infection and then decrease after 3 days.

They can be divided into anionic or cationic forms and based on their structure, and they can be either linear α-helices, peptides with a structure stabilised by disulfide bridges or peptides with glycine and/or proline residues [53,54,55,56]. Table 3 reports all the peptides involved in humoral response.

## 4. Experimental Advantages

The use of insects such as *G. mellonella* as an in vivo model follows the principle of the three Rs: replace, reduce and refine. According to this principle, animal testing should be minimised whenever possible in order to safeguard animals and reduce their suffering [2]. There are structural and functional similarities between the immune responses of insects and mammals. Both species show phagocytosis and the production of superoxide. These similarities can be observed in macrophage and dendritic cells in humans, and in plasmatocytes and granulocytes in insects. Insects have coagulocytes and oenocytes for coagulation, while mammals rely on a cascade activation of various factors [45]. C-reactive protein, a receptor homologous to mammalian cells, is able to recognise pathogen PRRs [51]. Today, *G. mellonella* larvae are also used to identify chemical compounds against pathogens, and in fact, this model has shown similar results compared with murine models [38]. To use a murine model, we still have to deal with many limitations concerning legal or ethical constraints, and obtaining the authorisation for mammalian studies can be a waste of time, while none of them are required when using *G. mellonella* larvae. The use of insects as an alternative to mammalian models does not require the presence of specialised personnel, although it is still necessary to train on the correct use and maintenance of the insects. The larvae can be stored in Petri dishes while mammals require a big space. Additionally, the short lifespan of the wax moth makes it suitable for high–throughput studies. Larvae can be incubated at 37 °C, allowing for the activation of temperature-dependent virulence factors in human pathogens. Several parameters can be used to evaluate the response of the larvae to infection. These include mortality, degree of melanisation, changes to haemocyte density and/or function, microbial burden, pupation, migration, gene expression and proteomic changes [5].

Compared to other types of invertebrates that are typically used, such as *D. melanogaster* (fruit fly), *G. mellonella* larvae are large enough to be easily handled by the operator [20]. The food they require is cheap and easily available.

While a normal laboratory rodent requires complex legislative housing and has very high costs, the experimental use as well as the buying and selling of *G. mellonella* larvae is not regulated by any legislation. In addition, it has very limited costs, and multiple replicates and statistically valid results are possible. However, as we will see later, this lack of housing also becomes a disadvantage in experimental analysis due to the absence of guidelines common to all laboratories [57].

## 5. Experimental Disadvantages

Like every model, *G. mellonella* also has its experimental limitations. Although the first draft of the *G. mellonella* genome (GenBank-NTHM01000000) has been deposited, not all immune proteins have yet been identified [22]. It is no coincidence that genome sequencing and studies of the immune response at the proteomic, epigenetic and transcriptional levels have opened up new fields of research. All this limits the presence of possible homologies with other organisms that can be used to compare the experimental data obtained between the model and the study subject [20].

All immune proteins have yet to be identified. This limits our knowledge about the larval immune system while maintaining the similarity already highlighted with mammalian immunity [21].

A further problem lies in the fact that different parameters are considered (mortality, melanisation, larval mobility, cocoon formation, quantification of haemocytes, concentration of haemolymph microorganisms) to analyse the course of an infection. This makes it impossible to compare the data with different laboratories [20].

Unlike mammalian models, insects do not possess an adaptive immune response; therefore, they do not produce antibodies but are limited to the production of proteins that confer only non-specific immunity [5,8,20,51,58].

## 6. *G. mellonella* as a Model to Study Fungi In Vivo

Human fungal infections have increased significantly in recent years. Treating these infections is extremely difficult due to multidrug-resistant (MDR) fungal strains that primarily infect immunocompromised patients. In addition, the limited availability of drugs and their toxicity constantly requires a study of new alternative molecules. The fungi most commonly associated with human diseases are *Aspergillus fumigatus*, *Candida albicans* and *Cryptococcus neoformans* [23,24,25].

*A. fumigatus* is the most lethal fungal pathogen in humans, with mortality rates of up to 90% [23]. *C. albicans* is the fourth most common cause of nosocomial infectious disease and the primary cause of systemic candidiasis, with high mortality rates [24]. *C. neoformans* is linked to illness and death in patients with weakened immune systems and those who have received transplants [25]. Over time, therefore, there has been an increased need for new models that can be used to study the pathogenic mechanisms of these microorganisms. *G. mellonella* is being exploited because it is an organism that can distinguish and identify different genera of pathogens while at the same time possessing an immune system, including some molecular homologies with that of humans. In addition, it can also be exploited to study the efficacy and toxicity of numerous antifungal agents [52].

Interestingly, the first work using *G. mellonella* as an in vivo model to study fungal species dates back to 2000. *G. mellonella* has been used to study many fungi, including *Aspergillus* spp., *Candida* spp., *Cryptococcus* spp., *Conidiobolus coronatus*, *Histoplasma capsulatum*, *Madurella mycetomatis*, *Malassezia* spp. and *Paraccocidioides brasiliensis* [59,60,61,62,63,64,65,66,67,68,69,70,71,72,73,74,75,76,77,78,79,80,81,82,83,84,85,86,87,88,89,90,91,92,93,94,95,96,97,98,99,100,101,102,103,104,105,106,107].

*G. mellonella* larvae have been successfully used to analyse fungal virulence, including toxin and enzyme production in vivo, providing an in-depth analysis of the processes involved in the establishment and progression of fungal pathogens. Virulence factors contributing to colonisation by pathogenic yeasts, including those belonging to the genera *Candida* spp., *Aspergillus* spp., *M. mycetomatis*, Mucormycetes and *Cryptococcus neoformans*, were examined in *G. mellonella*. In many cases, the larvae of *G. mellonella* have been used to test drugs and molecules with antifungal properties. Several antifungals, either alone or in combination, have been tested against different fungal species. In addition, *G. mellonella* has therefore also been used more to evaluate the effect of fungal extracellular vesicles on the immune system.

Categorising studies using *G. mellonella* larvae to assess virulence or drug efficacy is a challenging task. It is more appropriate to evaluate the work based on different fungal genera since some species have undergone extensive research in recent years [59,60,61,62,63,64,65,66,67,68,69,70,71,72,73,74,75,76,77,78,79,80,81,82,83,84,85,86,87,88,89,90,91,92,93,94,95,96,97,98,99,100,101,102,103,104,105,106,107].

### 6.1. Candida *spp.*

The capacity to induce systemic candidiasis and the high mortality rates mostly in immunocompromised patients make *Candida* spp. the most studied fungi.

*G. mellonella* larvae have been widely used as an in vivo model to study the virulence and mortality kinetics of systemic candidiasis in various *Candida* species [59]. In addition, researchers have evaluated the survival and the melanisation of the larvae in order to measure differences in virulence between reference strains and clinical isolates of *C. auris.* Further, the phenotypic switching in *C. tropicalis* variants crepe and rough has been studied [60]. Switching events in *C. tropicalis* affect biofilm development while sessile cells of distinct switch states may exhibit increased adhesion ability and enhanced virulence towards *G. mellonella* larvae. The phenotypic switching may cause higher levels of melanisation in larvae 24 h after infection, suggesting that the phenotypic switching of the yeast may produce structures that are detected differently in *G. mellonella* larvae [61]. The study revealed that surface pre-reacted glass-ionomer (S-PRG) is a bioactive filler generated through PRG technology and employed in different dental materials. The injection of S-PRG eluates into *G. mellonella* larvae did not have any toxic effects and helped protect them from experimental candidiasis. Furthermore, the eluate of S-PRG effectively hindered biofilm formation by *C. albicans*, *C. glabrata*, *C. krusei* and *C. tropicalis* while also providing protective benefits against experimental candidiasis in vivo for *G. mellonella*.

In one study, *G. mellonella* larvae were used to assess the effects of photodynamic therapy on experimental candidiasis and tissue responses to laser treatments [62]. The employment of *G. mellonella* larvae proved to be a beneficial model in examining light tissue penetration, thus improving the effects of antimicrobial photodynamic therapy. However, *C. albicans* showed greater virulence than *C. auris* in this system [63]. Similar results were reported in other research, showing the ability of *C. auris* to undergo filamentation in vivo with a mechanism comparable to that of *C. albicans* [64]. The dissemination of the fungus was evaluated through histological studies on larvae. Some authors have investigated the efficacy of a naturally derived polysaccharide called chitosan against aggregative (Agg) and non-aggregative (non-Agg) strains of *C. auris* in vivo. Chitosan reduced the fungal load and increased the survival rates of infected *G. mellonella*, while treatment alone was non-toxic to the larvae [65].

*G. mellonella* treated with 4-chloro-3-nitrophenyldifluoroiodomethyl sulfone (named Sulfone) showed the virulence reduction of *C. albicans* infections [66]. The correlation between extracellular virulence factors and the survival of *G. mellonella* larvae infected with clinical isolates of *Candida* spp. has been studied [67]. The results obtained showed that *C. albicans* and *C. glabrata* were more virulent, while *C. krusei* isolates were avirulent. The virulence of *C. parapsilosis* was an identified variable, and similar results were also observed in *C. albicans*, *C. tropicalis*, *C. glabrata* and *C. krusei* [68].

In another study, some researchers treated larvae of *G. mellonella* infected by *C. albicans* with the synthetic peptide T11F. This peptide has a sequence identical to a fragment of the constant region of human IgM. T11F has proved to be able to modulate the larvae immunity upon *C. albicans* infection, as determined by haemocyte analysis and larval histology [69].

Experiments with *G. mellonella* larvae injected by amphotericin B nanoemulsions, (NEA) have shown them to be extremely effective against candidiasis, in particular, anti-*Candica auris* action. Furthermore, NEA nanoemulsions can limit the acute toxicity typical of the amphotericin B [70]. The same authors have demonstrated antifungal activity in vivo on *G. mellonella* of nanoemulsions loaded with micafungin (NEM) in *Candida auris*. Although NEM did not show activity in planktonic cells, it exhibited action against biofilm and in the in vivo infection model [71].

A study using *G. mellonella* as an in vivo model was conducted to investigate fungal infections caused by *C. albicans* and *C. krusei* in association with implants. The research team utilised a planktonic and biofilm-implant model to test various antifungal drugs, namely amphotericin B, fluconazole and voriconazole, against the two species and assessed the fungal biofilm load on the implant surface. This investigation aimed to evaluate the efficacy of antifungal drugs in treating fungal infections associated with implants [72].

Some authors have highlighted the induction of the inflammatory response in *G. mellonella* in *C. albicans* strains with deletion of the α-subunit of F1Fo-ATP synthase compared to the wild-type strain [73]. It has been shown that Erg 6 overexpression acts as an effector of the Flo8 transcription factor and regulates biofilm and virulence in *G. mellonella* [74].

A comparison of two phenotypes of *C. parapsilosis* shows that the mean survival of larvae infected with Y132F-sinking isolates was significantly higher than that of the larvae infected with non-Y132F-sinking or non-Y132F-floating isolates [75].

The *Candida haemulonii* complex consists of rare multi-resistant yeasts that are often misidentified, while proving to be important healthcare-associated pathogens causing invasive infections. The echino-resistance pathway in *C. haemuloni* was investigated. Transmission electron microscopy analysis revealed changes in cell wall components, with a significant increase in cell wall thickness. The resistant strain also showed increased amounts of chitin (2.5-fold), a molecule localised in the cell wall. In addition, the resistant strain showed reduced virulence in the larval model of *G. mellonella* [76].

A maleimide compound [1-(4-methoxyphenyl)-1-hydro-pyrrole-2,5-dione, MPD] was identified as having potent antivirulence activity. Indeed, the survival time of *C. albicans*-infected larvae was significantly prolonged by MPD treatment [77].

In addition, phenyllactic acid (PLA), an important broad-spectrum antimicrobial compound, was investigated for its antifungal and antivirulence activities against clinical isolates of *Candida albicans*. The compound increased the survival rate of *G. mellonella* infected with *C. albicans* isolates [78].

The activity of *G. mellonella* antimicrobial anionic peptide 2 (AP2) against *C. albicans* has been examined by various microscopy and FTIR spectroscopy techniques. A decrease in fungal cell viability due to the action of the anionic peptide on the cell wall was observed with an increase in neo-formation and alteration of fungal wall proteins [79].

The pharmacokinetics of antifungal drugs can be studied in *G. mellonella* models, including the study of drug uptake and distribution in the haemolymph, drug metabolism and half-life [80]. In order to assess whether the FKS1R658G mutant in *C. parapsilosis* confers resistance to echinocandin and causes therapeutic failure of echinocandin, *G. mellonella* larvae were infected with both the parental and the mutant strain. The fungal load was assessed 24 h after infection. As expected, the larvae infected with the mutant strain had a significantly higher fungal load than the parental strains treated with caspofungin and anidulafungin. Unexpectedly, micafungin was also ineffective against the larvae infected with either the parental strain or the mutant strain carrying FKS1R658G. This may indicate that either the micafungin concentration needed to be higher than that used in Galleria or that micafungin is metabolised more rapidly in Galleria and, therefore, does not show efficacy [81].

One effective strategy to combat drug-resistant pathogens comprises the administration of molecules restoring fungal susceptibility to approved drugs. 1,4-benzodiazepines selectively potentiate different azoles, while they do not have the same effect on different antifungals. The potentiators were not toxic to *C. albicans* in the absence of fluconazole, but they inhibited the virulence associated with the filamentation of the fungus. Researchers found that the combination of the potentiators and fluconazole significantly enhanced host survival in a *G. mellonella* model of systemic fungal infection [82]. These molecules were also able to inhibit filamentation (a virulence-associated trait), as tested by Shapiro [83]. Exposure to *C. albicans* extracellular vesicles has been shown to have a protective effect on *G. mellonella*, reducing insect mortality following the fungus against infections [84].

### 6.2. Malassezia *spp.*

*Malassezia* spp. is an opportunistic pathogen associated with various human and animal skin diseases, such as pityriasis versicolor, psoriasis and seborrheic dermatitis.

The experiments conducted on *G. mellonella* using *Malassezia furfur* and *M. pachydermatis* found that the larvae’s survival is affected by the incubation temperature after infection. The infected larvae were incubated at two temperatures, 33 °C and 37 °C. At 37 °C, *M. pachydermatis* was slightly more virulent and had a higher fungal load than *M. furfur*, which was more virulent at 33 °C despite having a lower concentration. The studies showed that both larval mortality and melanisation were dependent on the *Malassezia* species, inoculum concentration and temperature [85,86].

### 6.3. Cryptococcus *spp.*

Cryptococcal meningitis is a severe infection of the central nervous system caused by encapsulated yeasts, specifically *C. neoformans* and *C. gattii*. Increased resistance to fluconazole has been observed with variable virulence. Comparative studies of virulence in *G. mellonella* larvae between naturally fluconazole-resistant strains and resistance-induced strains have shown that the latter are less virulent than the original susceptible strains [87]. In *C. neoformans*, the analysis of gene expression during infection with Galleria revealed a small number of different genes involved in the ROS response [88]. *G. mellonella* larvae infection model was used to evaluate the in vivo effects of hydroxychloroquine (HCQ) and itraconazole (ITR) on *C. neoformans*. In comparison to ITR alone, the combination of HCQ and ITR treatment increased the survival of larvae while decreasing the fungal burden of infected larvae [89]. An antimicrobial peptide with 100% homology to *Drosophila virilis* (DvAMP) has significant in vivo therapeutic effects on *G. mellonella* larvae since it reduces mortality and fungal load in *C. neoformans* larvae, suggesting that this peptide may be a promising antifungal candidate for the treatment of cryptococcosis [90]. Similar results were also reported using vitamin D3 (VD3) [91]. At last, it has been reported that organoselenium is able to reduce the burden of *C. neoformans* in vivo, as well as inhibit specific virulence factors [92].

### 6.4. Aspergillus *spp.*

*Aspergillus* spp. is one of the main pathogens causing diseases in immunodeficient subjects. *A. fumigatus* is one of the most critical fungal pathogens for which innovative antifungal treatment should be prioritised. *G. mellonella* larvae were used as a model to screen antifungal drugs against triazole-sensitive and triazole-resistant *A. fumigatus* infections. The model includes a statistically powerful quantitative and longitudinal analysis of *A. fumigatus* load to optimise the preclinical antifungal screening. The authors show that bioluminescence imaging is a more reliable, sensitive and quicker method for quantifying fungal load. This method can not only detect the treatment effects for both susceptible and triazole-resistant infections, but it can also improve the translatability of in vitro antifungal screening results to in vivo confirmation in mouse and human studies [93].

Specialised metabolites, such as the ergot alkaloid fumigaclavine C in *A. fumigatus*, have been found to increase fungal virulence in *G. mellonella*. In this study, the pathogenic potential of three recently discovered Aspergillus species that can accumulate high concentrations of lysergic acid α-hydroxyethylamide (LAH) was investigated in *G. mellonella*. The results showed that *Aspergillus leporis* was the most virulent, followed by *A. hancockii*, while *A. homomorphus* had a very low pathogenic potential. Two fungi-producing ergot alkaloids, and which were not previously known as opportunistic pathogens, can infect larvae. In at least one of the species, the presence of an ergot alkaloid increases the virulence of the fungus [94]. A prolonged subculture of *A. fumigatus* on agar generated from *G. mellonella* altered the virulence in larvae [95]. *A. fumigatus* mutants with defects in melanin biosynthesis cause an increase in larval mortality after infection, highlighting the importance of studying the innate immunity of the insect [26].

The combination of echinocandins with azoles is an attractive alternative option for the treatment of invasive aspergillosis due to azole-resistant *A. fumigatus* strains. The combination of caspofungin (CAS) with either voriconazole (VRZ) or posaconazole (PSZ) on *G. mellonella* shows that the combination of caspofungin with azoles is a promising alternative for the treatment of azole-resistant strains of *A. fumigatus* [96].

### 6.5. Other Genera

Paracoccidioidomycosis is an infection caused by *Paracoccidioides* that usually affects the lungs and skin. The symptoms usually get worse in people with a weakened immune system.

*G. mellonella* is a suitable model for studying the virulence mechanisms of *Paracoccidioides brasiliensis* due to its advantage of faster isolation of fungi (4 days) as compared to mice (30 days) [97]. *G. mellonella* larvae were also used to study the role of a lectin from *P. brasiliensies* and its role in virulence, showing that the fungus is less virulent in the absence of this lectin [98]. It has been tested on *G. mellonella*, a peptide with an affinity for the PbDrk1 protein of *P. brasiliensis*, which probably plays a crucial role in morphology and virulence. This peptide may increase the effects of certain antifungal agents, and it was also evaluated for its efficacy in vivo. It has been demonstrated that this contributes to the increased survival rates of larvae [99].

Similar results were documented in vivo regarding *Fusarium keratoplasticum* and *F. moniliforme*, filamentous fungi prevalent in the environment that can cause mycosis in both animals and plants [100].

Infection of the larvae with *F. oxysporum* results in nodulation and melanisation, ultimately leading to larval death due to over-colonisation [101].

*G. mellonella* has been used to study the virulence of more complex fungal species, such as *dematiaceous* fungi, *mucormycetes* and *Penicillium marneffeis* [102,103,104,105]. The two most common causing agents of black-grain eumycetoma are *Madurella mycetomatis* and *Falciformispora senegalensis*. Since grains cannot be formed in vitro, in vivo models are needed to study grain formation. Naphthazarin (5,8-dihydroxy-1,4-naphthoquinone) was identified as a considerably active antifungal compound against *Madurella mycetomatis* (IC_50_ =1.4 μM), while it showed reduced toxicity to *G. mellonella* larvae, which is a well-established in vivo invertebrate model for mycetoma drug studies [105].

The invertebrate *G. mellonella* is employed to induce grain formation in vivo for *F. senegalensis*. The grains that developed in larvae were analogous to those that formed in patients, thus indicating the viability of this model for monitoring grain formation [106].

The virulence and melanisation in *G. mellonella* models for *Fonsecaea monophora*. were observed, and the death rates of infected larvae were positively related to injected concentrations of fungus [107].

## 7. Conclusions

The significance of *G. mellonella* in fungal pathogen experimentation is apparent. *G. mellonella* is a valuable model due to its advantageous experimental features, compliance with the three Rs, ease of use and affordability. Its short life cycle also allows for favourable testing times. Furthermore, its similarities with the human immune system make it an optimal experimental tool. However, despite its advantages, there are also some drawbacks to consider. The main problem is the lack of guidelines regulating its use, which makes it impossible to compare experimental data from various laboratories. Furthermore, the first draft of the *G. mellonella* genome has been deposited, and not all the proteins involved in the immune response have been identified. Nevertheless, despite the limitations of using larvae as an in vivo model, it remains a valuable tool for studying fungal infections due to its numerous advantages. To date, over 700 studies have been conducted on *G. mellonella* larvae to test the pathogenicity of fungi, despite the aforementioned limitations. Comparative studies between murine and *G. mellonella* models have demonstrated their reliability and comparability with other experimental models currently in use. These studies analysed virulence as well as immune response and the effect of antimicrobials.

## Figures and Tables

**Table 1:** In vivo infection models with invertebrates.

Species	Size (mm)	Special Equipment for Organ Isolation	Special Handling Technique for Administration	Route of Administration/Accuracy of Administered Dosage
*Drosophila melanogaster*	1–3	Required	High	Oral, injection to dorsal surface, not accurate
*Bombyx mori*	50–60	Not required	Low	Oral, injection to dorsal surface: intra haemolymph, intra-mid-gut/accurate in the case of injection
*Galleria mellonella*	20–40	Not required	Low	Oral, topical, injection to ventral surface/accurate in case of injection

**Table 2:** Different types of haemocytes and their functions.

Type Cells	Description	Functions
Prohaemocytes	Progenitor cells	Differentiate into several cell types
Plasmatocytes	Most abundant, present lysosomal enzymes in the cytoplasm	Produce antimicrobial factors, participate in phagocytosis
Granulocytes	Small nucleus, granules in the cytoplasm	Participate in phagocytosis directly in the encapsulation process
Spherulocytes	Very large, highly polymorphism, large granules in cytoplasm	Transport and secrete several cuticular components
Oenocytoids	Round shape, small eccentric nucleus, homogenous cytoplasm, microtubules, ribosomes	Involved in the melanisation pathway to secrete extracellular nucleic acid, involved in pathogen sequestration; coagulation activation
Coagulocytes	Spherical cell, large nucleus, hyaline cytoplasm	Involved in haemolymph coagulation, encapsulation

**Table 3:** Components of humoral response in *Galleria mellonella*.

AMP Anionic		References
AP1	Reduced phenoloxidase activity in haemolymph	[52,53,54]
AP2	Reduced metabolic and fungistatic activity towards *C. albicans*; synergistic action with lysozyme	[55,56]
AMP Cationic		
Linear α-helical	Peptides without cysteine residues among them, cepropins and moricins are active against bacteria and filamentous fungi	[54]
Peptides with disulfide bridges	Peptides contain three or four disulfide bridges, gallerimycin and galiomycin, which are defensive peptides against fungi binding to hydrophobic component such as β-1,3 glucan, LPS and LTA	[56]
Proline- or glycine-rich residues	Peptides, such as Gm proline-rich peptide 1, inhibit growth against yeast and glycin-rich residues, such as gloverin, which inhibit the synthesis of membrane proteins in bacteria	[56]
Lytic Enzyme		
Lysozyme	Inhibits *C. albicans* growth in a dose-dependent manner with the reduction of metabolic activity and shows fungicidal activity	[55]
Opsonin		
apoLp-III	Binds to hydrophobic components, such as β-1,3 glucan, LPS and LTA, inducing apoptosis and phagocytosis, involved in detoxification. Increase in haemolymph antibacterial activity and the production of superoxide. Synergistic activity with lysozyme toward Gram-negative bacteria	[54]
PGRPs	Peptidoglycan-binding proteins induce hydrolysis	[52]
Haemolin	Haemolin is a member of the immunoglobulin superfamily, increase in the production of haemolin after infection with bacteria and viruses	[49,52]
